# Presenteeism, Overcommitment, Workplace Bullying, and Job Satisfaction: A Moderated Mediation Relationship

**DOI:** 10.3390/ijerph17228616

**Published:** 2020-11-20

**Authors:** Francisco Rodríguez-Cifuentes, Samuel Fernández-Salinero, Juan Antonio Moriano, Gabriela Topa

**Affiliations:** 1 Psychology Department, Universidad Rey Juan Carlos, 28933 Madrid, Spain; francisco.rcifuentes@urjc.es (F.R.-C.); samuel.fernandez@urjc.es (S.F.-S.); 2 Department of Social and Organizational Psychology, National Distance Education University (UNED), 28040 Madrid, Spain; jamoriano@psi.uned.es

**Keywords:** presenteeism, overcommitment, workplace bullying, personal bullying, work-related bullying, job satisfaction, economic stress

## Abstract

Presenteeism is a hazardous behaviour that may have personal and organizational consequences. The main objective of this research was to investigate the relationship between presenteeism and job satisfaction and evaluate the role of overcommitment as a mediator and the role of work-related and personal bullying as moderators in these relationships. Results from 377 subjects showed that presenteeism and overcommitment are positively related to job satisfaction, with overcommitment being a mediator in the relationships. These relationships are moderated by work-related bullying but not by personal bullying. The findings are discussed, and implications, future research pathways, and limitations are noted.

## 1. Introduction

The COVID-19 outbreak has shown the fragility and precariousness present in some economic sectors. In addition to the health crisis itself, work conditions have changed dramatically, affecting workers’ physical and mental health. The predicted global economic crisis may prompt inefficient and inadequate decisions from both organizations and workers, and the field of occupational health psychology may encounter new challenges ahead.

For years, organizations have focused on the improvement of their results by optimizing the efforts of their workers. The intuitive notion is that motivated and committed workers will obtain better results than those who are not committed. This is the reason behind the huge amount of tries from companies to provide incentives to their people, not only extrinsic rewards, like higher salary, but other kinds of rewards that are able to influence the efforts of their workers, like being a valuable member of a team, performing interesting tasks or knowledge sharing [1].

These actions have turned out to be useful in not only augmenting the benefits of the organization but also creating better workplaces where people feel comfortable with their partners and are able to develop their skills. Several investigations have also noted the positive impact of individual variables, such as job satisfaction [2] and self-efficacy [3], which are, of course, an advantage to the organization as a whole.

In fact, the importance given to the employees’ well-being has increased in the past decades, but unfortunately, this seems to be insufficient. On the one hand, not all firms have the means—or at least they do not put them into action—to focus on wellness within their organization, as they are overwhelmed by many objectives that they want to accomplish. Regrettably, these companies ignore the benefits they could obtain from implementing an employee-oriented approach apart from customer orientation.

On the other hand, although there have been attempts to create better workplaces and obtain job satisfaction within the organization, some negative aspects seem difficult to remove. Some undesirable social dynamics keep happening within the organization; one proof of this is the amount of investigations on mobbing [4,5,6]. This undesirable situation is even more dangerous right now due to the found relationship between economic stress and workplace bullying [7]. If that is not enough, finding the best way to motivate people is not easy at all due to individual differences in various aspects such as “what is motivating” or “how is motivation managed.” Therefore, the idea previously referred to as the relationship between motivation and performance could perhaps be wrong or at least not entirely true.

An area that has received attention in the last decades is the presence or absence of jobs. Traditionally, absenteeism has been reported as a symptom of low motivation, and its relationships with desirable outcomes such as satisfaction or performance are well known. Nevertheless, some previous studies have discussed the negative consequences of presenteeism, which receives much less attention from companies [8], and its effects may be more disastrous than those of absenteeism. This behaviour could reflect a misunderstanding about how people should act properly or, seen in another way, a pernicious management of motivation. Nowadays, the serious consequences that this kind of behaviour seem even clearer, not just for the achievement of objectives, but also for the wellness of coworkers.

In order to better understand the existing relationship between these variables, our research tries to clarify how presenteeism and job satisfaction are related. Nowadays, job satisfaction is one of the most studied variables due to its importance within the organization, and understanding how it is related to other variables is our focus. The main objective of this research is to evaluate the influence of presenteeism on job satisfaction mediated through overcommitment and moderated by bullying in the workplace.

### 1.1. Presenteeism

According to Lohaus and Habermann [9], presenteeism has been studied using two principal approaches. From the American perspective, this phenomenon is understood as the loss of work productivity due to a person’s health problems. On the other hand, from the European viewpoint, this variable focuses on job attendance even when workers’ health status is not ideal, and they are better off staying at home. In the latter perspective, which we adopt in this paper, the interest is to discover both the antecedents and consequences. Other researchers have made a broader definition, focusing on being present but functionally absent, not only limited to illness [10]. Although we found this approach to be interesting, as both result in productivity loss, we choose the health-related perspective due to its fitness to occupational health psychology [11]. The integrative factor in these definitions is the relationship with productivity. Indeed, this concept has been understood as synonymous to individual work performance.

Presenteeism could be conceptualized as an effort made by workers, but some research studies have understood this process, as well as absenteeism, as a counterproductive work behaviour, highlighting its negative consequences [12,13,14]. The theorized reasons for its occurrence are varied, which can be categorized as personal and contextual variables. Examples of the first category are suffering a psychiatric disorder [15], educational level, age, and being a parent [16], while leadership [17], uncertainty over promotion opportunities [18], work environment, and time pressure [19] have been proposed as situational antecedents. It could seem that based on the variables listed, all the causes may root from negative aspects, but it is not valid, as the variables of work joy or self-confidence and self-image could be the reason to show up ill at work [20].

The usually listed consequences include aggravated health status [21], poorer performance [22], and job dissatisfaction [23]. As noted, the negative impact of presenteeism is not only with the individual but with the whole organization. An important aspect is that presenteeism is harder to measure [24], which can perpetuate the negative outcomes. Once again, it is necessary to point out that consequences are not always considered negative (i.e., workers report occasions where presenteeism had positive consequences, like being beneficial to their recovery from illness [20], or their productivity increases in such situation as compared with being absent from work [25]).

### 1.2. Overcommitment

The ERI (effort–reward imbalance) model [26] states that nonreciprocity between high efforts and low rewards generates stress. The model emphasizes workers’ subjective perception of working conditions, disentangling an extrinsic component (effort–reward ratio, based on the calculation of “workers’ benefits”) from an intrinsic component (overcommitment).

The ERI model has been found to be useful in explaining work realities in different countries and sectors [27,28,29,30], and its components have been reported to be linked to variables such as job satisfaction, occupational well-being, turnover, dedication, and absorption [31].

Overcommitment has been defined as “a set of attitudes, behaviours and emotions that reflect excessive striving in combination with a strong desire to be approved of and esteemed” [32] (p. 55). Allen and Meyer [33] conceptualized a three-component model of organizational commitment: affective, normative, and continuance. From this perspective, overcommitment is closer to affective commitment, which is defined as an “employee’s emotional attachment to, identification with, and involvement in the organization” [34] (p. 67). Several studies have related this kind of commitment to important variables, such as intention to quit [35], burnout [36,37], work performance, citizenship behaviours [38], and job satisfaction [39]. Affective commitment has also been found to be negatively linked to bullying [40], and several studies have proposed commitment as a mediator in the relationships that job satisfaction has with other variables [41,42].

Siegrist and Li [43] stated that overcommitted people are expected to exhibit an intense need to be in control and excessively engaged, which lead to an inadequate coping style with the demands of the organization.

One important relationship has been found between overcommitment and burnout [44], which is related to negative conditions in companies, such as absenteeism, decreased job satisfaction, and lower productivity [45]. This results contrast with those of other studies, other than the association between highly committed workers and less turnover intention [46], which reinforces the idea that overcommitment could be a wrong way of understanding workplace reality: they are workaholics.

### 1.3. Job Satisfaction

Job satisfaction has turned out to be one of the variables given the most attention, not just in the academic field but also in companies. Eventually, people’s opinions are considered, whether they are part of the company or not. This way, customer service becomes the pillar of modern firms, but making workers happy is probably even more important. Several studies have related job satisfaction to desirable outcomes in companies, such as contextual performance [47], self-efficacy, motivation, and commitment [48].

Despite this growing interest in this variable, its definition is not easy. In this paper, we adopt the concept as a positive affective response to a job [49], which is affected by both contextual and individual variables [50]. This perspective is consistent with the ERI model, in which both kinds of variables are considered.

This idea makes organizations, at least partly, responsible for the well-being of their workers. The traditional transactional relationship may be insufficient nowadays, and different approaches that pay attention to other workers’ needs seem crucial to organizations’ survival.

### 1.4. Workplace Bullying

Violence and aggressiveness have been present throughout human history. Nowadays, some authors state that we are living in more peaceful moments of our existence, but regrettably, abuse and mistreatment have not disappeared from our lives.

There are some ambits where this kind of undesirable behaviour is especially investigated: school, gender violence, racism, and of course, workplace. In organizations is frequently named mobbing; however, to not stand out distinction with other types of bullying (they are all reprehensible) and maintain consistency among terminologies, we prefer to use workplace bullying in this paper, which has a synonymous meaning in the literature [51].

One of the main traits of this work-related phenomenon is that violence is presented not psychically but, in a slight way, using gossips or jokes to create a conflictive environment, damaging people affectively. As León-Pérez et al. stated [52], workplace bullying can be considered a stressful situation that hits out at people’s needs, which can end up in the appearance of negative health conditions [53]. Again, subjectivity is vital since the “definitional core of bullying at work rests on the subjective perception made by the victim that these repeated acts are hostile, humiliating and intimidating and that they are directed at himself/herself” [54] (p. 249).

Workplace bullying has repeatedly been related to low job satisfaction, turnover intention [55,56], more sick leaves, absenteeism [57,58], and lower dedication [59], and its negative relationship with job satisfaction has been noted for years [60]. Workplace bullying has been reported as an exacerbator (moderation effect) of the effects of job demands on physical exhaustion, depression, and uncertified absenteeism [61]. The negative consequences are not just associated to the work environment, but also there are impacts outside work (e.g., an increment of family conflicts [62] or increased costs to society due to sickness and job losses) [63]. Conversely, fostering a teamwork environment could have positive consequences, including reduced presenteeism [25].

Factor analyses have found consistent results for both victims and perpetrators regarding two factors: person-related and work-related bullying [64].

### 1.5. Hypothesis

The main aim of the present research is to analyse the impact of presenteeism on job satisfaction. In addition, we propose overcommitment as a probable mediator and bullying playing a probable moderation role in relationships.

**Figure 1:**
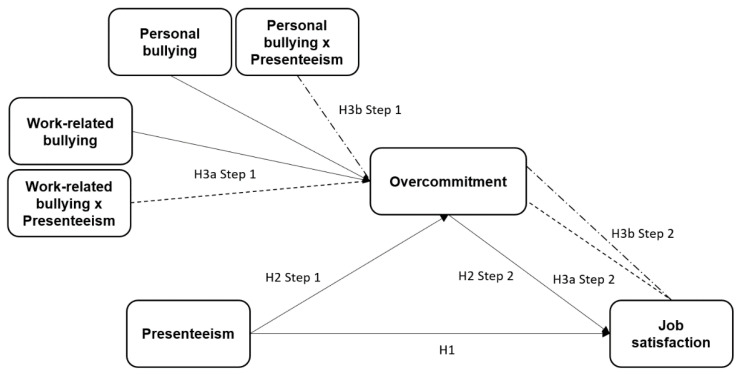
Graphical model of the hypotheses where the relationships between variables are shown.

First, we propose that presenteeism has a negative and statistically significant relationship with job satisfaction (H1). Second, we propose that this relationship is negatively mediated by overcommitment (H2). The last hypothesis tested is about the moderation effect that work-related bullying has on the aforementioned relationships (H3), specifically work-related bullying (H3a) and personal bullying (H3b). Based on the reviewed literature, we expect this moderation to have a negative effect as well. The hypotheses are shown in Figure 1.

## 2. Materials and Methods

### 2.1. Sample

To achieve our objectives, a homogeneous purposive sampling was used. Data were collected from small- and medium-sized organizations from different regions in Spain and economic sectors (services, HR consultancy firms, and production) in an attempt to represent the business reality of the country, and these organizations were associated with the university through research agreements. The organizations were informed about the purpose of the research and the time required to answer the questionnaire. The companies that agreed to collaborate were sent the questionnaires (response rate: 33.52%), and they distributed them to their employees, who also had to agree to collaborate with the study.

The sample was composed of 377 subjects who participated in our research and agreed to collaborate voluntarily. There were 48.3% women and 51.7% men. The mean length of time working in the company was 12.59 (9.6 SD) years, while the average age was 41.46 years old (9.5 SD). Regarding academic level, 8.8% of the subjects had basic education, 8.8% finished high school, 18% had vocational training, and the rest finished university studies: 48% university degree, 15.6% master’s degree, and 0.8% Ph.D. Regarding contract type, in our sample, 66.8% were temporary workers, and 33.2% had an indefinite contract.

The study was approved by National Distance Education University’s bioethics committee in January 2020 (ethical approval code number 20012020). The ethical and legal aspects were approved. The subjects received a link to the questionnaire, where instructions were given. They were informed that the questionnaire did not collect personal data and that their participation was strictly voluntary. Once the questionnaires were completed, data were analysed with SPSS v.24.We used Hayes MACRO for SPSS v.24; specifically, model 9 was used.

### 2.2. Instruments

The questionnaire used in this research was composed of instruments noted below. The selection of the instruments was based on bibliographic research, fit with the followed definitions and approaches, and their previous validation and use in investigations.

*Presenteeism*. The Stanford presenteeism scale [65] was used. This instrument is formed by six items (SPS−6; e.g., “At work, I was able to focus on achieving my goals despite my health problem”) and is presented as a five-point Likert scale, from 1 (strongly disagree) to 5 (strongly agree). Cronbach’s α was 0.924. The single-factor confirmatory factor analysis (CFA) model for presenteeism fit the data quite well, except for the root mean square error of approximation (RMSEA) value (χ^2^_(13)_ = 64.891, *p* < 0.001, comparative fit index (CFI) = 0.968, RMSEA = 0.138, standardized root mean square residual (SRMR) = 0.026).

*Overcommitment*. A shorter version of the overcommitment scale of the ERI questionnaire [19] was used. This recommended version [66] is composed of five items from the subscale “personal inability to withdraw from work obligations” (e.g., “I get easily overwhelmed by time pressures at work”) and one item from the subscale “impatience and disproportionate irritability (“If I postpone something that I was supposed to do today I’ll have trouble sleeping at night”). In this study, we transformed the four-point Likert scale items into a Likert-type scale from 1 (strongly disagree) to 5 (strongly agree). Cronbach’s α was 0.877. The single-factor CFA model for overcommitment fit the data satisfactorily, except for the RMSEA value (χ^2^_(12)_ = 49.037, *p* < 0.001, CFI = 0.967, RMSEA = 0.109, SRMR = 0.036).

*Workplace bullying.* For the assessment of this variable, we used the reduced Spanish version of the Negative Acts Questionnaire (NAQ) [60], validated by Moreno, Rodríguez, Martínez, and Gálvez [67]. This scale is formed by two subscales: “work-related bullying” and “personal bullying”. Regarding the first subscale, in our sample Cronbach’s α was 0.837, which is a good level of reliability. The questionnaire is composed of eight Likert scale items (e.g., “My opinions or views have been neglected”), whose values range from 1 (never) to 5 (daily). On the other hand, the personal bullying subscale has the same form as the other subscale but is composed of six items (e.g., “Gossip and rumours have been spread about me”). Cronbach’s α was 0.793. The global reliability of the instrument (both subscales) was 0.881. The two-factor CFA model for workplace bullying showed an acceptable fit to the data (χ^2^_(33)_ = 251.569, *p* < 0.001, CFI = 0.909, RMSEA = 0.082, SRMR = 0.059).

*Job Satisfaction*. To measure this factor, we used the Brief Index of Affective Job Satisfaction (BIAJS) designed by Thompson and Phua [68], which showed Cronbach’s α = 0.870 in our sample. This seven-item instrument (e.g., “Most days I am enthusiastic about my job”) presents three distractors (e.g., “My job is unusual”). The instrument is a five-point Likert scale, from 1 (strongly disagree) to 5 (strongly agree). Confirmatory factor analysis of the single-factor model showed an acceptable fit to the data (χ^2^_(8)_ = 3.411, *p* < 0.001, CFI = 0.998, RMSEA = 0.043, SRMR = 0.012).

## 3. Results

To assess our hypotheses, we used the PROCESS macro [69], specifically Hayes model 9, which was developed as a moderated mediation model and allows two moderating variables. The PROCESS macro extracted 1000 random samples from the original data used through a bootstrapping procedure. Before testing the model, we also carried out a correlation analysis among the variables and studied possible collinearity problems between the variables. All Variance Inflation Factor (VIF) values were lower than 5, and their tolerance values were higher than 0.2, so there was no collinearity problem. The results are shown in Table 1, where you can see that there is no collinearity problem (VIF values < 5; besides, their tolerance values were higher than 0.2), and as expected, the significant relationships were in the predicted directions.

### 3.1. Mediation Analysis

First, we assessed the direct effect of presenteeism on job satisfaction. We found that this relationship was positive and statistically significant (B = 0.34, SE = 0.46, 95% CI [0.25; 0.43], *p* < 0.001). Furthermore, overcommitment is related to job satisfaction in the same terms (B = 0.14, SE = 0.05, 95% CI [0.04; 0.23], *p* < 0.05).

### 3.2. Moderation Analysis

Second, we tested whether overcommitment (M) is moderated by both types of bullying, work-related (W) and personal (Z), when there is presenteeism (X). Results supported this hypothesis in the case of work-related bullying (B = 0.39, SE = 0.13, 95% CI [0.13; 0.65], *p* < 0.05), but were not statistically significant in the case of personal bullying (B = −0.14, SE= 0.21, 95% CI [−0.55; 0.27], *p* > 0.1). Notably, at lower values of bullying, overcommitment is lower (the effect is negative; coefficients < −0.17), while higher values of work-related bullying have a positive effect on overcommitment (coefficients > 0.15). Therefore, the moderation model is supported at lower (at 16th percentile = 1.125) and higher values (at 84th percentile = 2.125) of work-related bullying (*p* < 0.05).

### 3.3. Moderated Mediation Analysis

**Figure 2:**
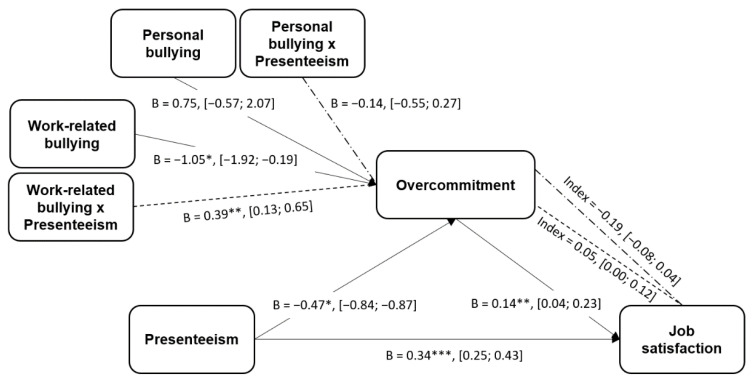
The statistical model where coefficients and effect sizes may be observed. (*Note*: * *p* < 0.05, ** *p* < 0.01, *** *p* < 0.001).

In the end, we can affirm that our moderated mediation model is partially statistically confirmed. Presenteeism (X) has an effect on job satisfaction (Y) through overcommitment (M), and this relationship is moderated by work-related bullying (W; B = 0.05, SE = 0.02, 95% CI [0.00; 0.12]) but not by personal bullying (Z; B = −0.02, SE = 0.03, 95% CI [−0.08; 0.04]). Notably, at lower values of work-related bullying, job satisfaction values are lower. At the 16th percentile level, the effect on job satisfaction is statistically significant (<−0.2), except when personal bullying values are higher (B = −0.03, SE = 0.02, 95% CI [−0.07; 0.00]). All the analyses performed are shown in Figure 2, including the obtained index and the confidence intervals.

## 4. Discussion

The main aim of the current research was to evaluate the impact of presenteeism on job satisfaction, taking into consideration the probable mediation role of overcommitment and the probable moderation role of bullying in these relationships.

As expected, presenteeism is found to be related to job satisfaction, but not in the predicted orientation. Contrary to the initial hypotheses, the relationship is positive, which means that better attitudes through presenteeism are associated with job satisfaction. This result reflects that job satisfaction is not an easy variable to explain. One possibility that could explain this surprising result is that people’s presenteeism is due to their own willingness, diminishing the negative aspects of this behaviour and having the perception that they love their job because they work even when not yet fully recovered from their illness. This interpretation would be valid in explaining the positive relationship between overcommitment and job satisfaction; assuming this origin from the individual perception, presenteeism allows understanding overcommitment as work fulfilment and as capable of leading to job satisfaction. This explanation is linked to previous reports about how workers understand presenteeism [20]; while from a managerial perspective this could mean a decrease in productivity, people may understand presenteeism as the ideal way to cope with illness, which could increase their job satisfaction. These results would fit the results of other studies that highlight the importance of the attributions of voluntariness in job satisfaction [70,71]. Another possibility (and a limitation of the research) is that not only presenteeism is relevant to the interpretation of information, but the general health status [72]. If people score high on general health status or, from a different perspective, their illnesses are not a big issue, presenteeism would be compatible with high job satisfaction.

Hypothesis 2 is accepted in that overcommitment appears to be a mediator variable in explaining job satisfaction, which is consistent with previous studies [73,74]. The unusual aspect found here is the negative relationship between presenteeism and overcommitment, but this is not the first investigation that has found such inverse relationship [75]. A tentative explanation is that even though both behaviours share a probably misunderstood commitment, presenteeism is related to a sense of being able to do the job when individuals should not, while overcommitment is related to their extra effort due to not being able to cope with the requirements of the job. Again, the individual’s perception is key to understanding the underlying relationships among variables.

Finally, hypothesis 3 is partly confirmed: work-related bullying has an impact on the proposed model but not personal bullying. This makes sense in the previous discussion: overcommitment is related to the individual’s interpretation of not being able to accomplish properly the tasks, and extra efforts are needed to overcome the situation, and work-related bullying focuses on underestimating the individual’s work. By examining the results better, it is found that the direct interaction between work-related bullying and overcommitment is negative (it generates negative feelings that reduce commitment) [76,77], but interaction with presenteeism minimizes this effect. As noted, the final impact of bullying on satisfaction is positive, which fits the results of previous investigations on task conflict and job satisfaction [78]. Understanding workplace bullying as a stressor but being able to motivate people could even have other positive effects in innovation development [79]. Although this “benefit” of bullying, it is needed to reinforce the idea that this process is totally undesirable and that there are differences among people [80], not everyone can cope with workplace bullying. Not all coping behaviours have the same effect [81].

To sum up, we found that (a) presenteeism could be positively related to job satisfaction, (b) overcommitment is a mediator variable in this relationship, and (c) work-related bullying, but not personal bullying, moderates all these relationships.

As noted throughout the document, individual perceptions are important in order to understand the variables studied and their relationships. It seems that companies have a responsibility to develop a better belief of what is expected and desirable from their workers. This implies shifting the cultural perspective toward a worker-centred vision, preventing bullying [82], and promoting autonomy [83] and mutual recognition of respect among all members [84], which tends to better workplace attitudes. The point of view of perpetrators should be given attention to identifying the factors that generate this process. In this regard, workplace bullying must be analysed as one more work stressor [85] and productivity destroyer [86]. An effective management is vital to reducing these kinds of processes, as well as presenteeism. Achieving this objective would help to reduce risks related to human resources and promote sustainable organizations [87]. This aim seems even more important nowadays with the pandemic scenario: providing an optimal approach for workers to work, including showing the suboptimal consequences of presenteeism [88], could benefit not just the organization but even societies. Safety at work, in its expanded meaning, has an impact on all of us.

Some limitations should be considered. First, the research design is cross-sectional, which may lose precision in the results; longitudinal research is appropriate to validate them. Second, the information was collected with the use of self-reported measures, and it may bias the results. Additionally, this study only represents a selection of variables that have effects on job satisfaction, but the relationships studied here may be affected by some other variables, like social support and leadership style.

One open question, despite the broad investigation, is how people get motivated. Our research reflects a paradoxical result where a negative process could have positive effects in the end, which leads us to propose an analysis of relationships with variables like coping mechanisms and resilience. Unfortunately, eradicating bullying could be ideal but not easy to achieve right now, and at least being able to minimize their potential hazards could be useful. Hopefully, this could reduce these social processes by eliminating the damage they seem to have.

Future research should go deeper into the effect of other sociological variables that have been linked to the variables examined in this paper, such as hierarchical level or contract type [89] or being a foreigner [90], without forgetting to integrate the results with modern realities, like social virtual worlds [91]. It is an organization’s responsibility to better know why behaviours like presenteeism are observed in their workers, as well as their consequences not just on job satisfaction but also on their social status within the organization, their financial wealth, and their health. These consequences are meant to be addressed not individually but as a group and even as a whole society. A current example is that, with the fear of COVID-19, companies can acquire a bad reputation for having their employees working with symptoms; it seems illogical that a health crisis had to occur to realize how negative it can be to show up at work without being at full capacity.

A possible way out in many cases could be digitalization, which is an open front of the 21st-century economy, but it is necessary to understand that workers’ behaviours will adapt to this paradigm according to the behaviour of their organizations: providing an adequate cultural vision or information and opportunities to follow technological advances [92] is crucial. This dynamic seems to be precipitated even more in this global pandemic situation. Behaviours that could be understood as an example of high commitment will probably be frowned upon hereinafter, and the meaning of responsibility will change: it makes no sense to reward unuseful efforts, and cultural values may be behind this inefficiency. Staying working until the boss has left is the usual behaviour for many workers due to the subjective, and not objective, reward system present in work teams. It is necessary to prioritize efficiency over quantity now more than ever, taking into account that showing up at the office with a fever could cause paralysation of the economic activity of the firm. We count on the technologies needed to transform most jobs into a non-presential exercise, but they are not enough to change the frame.

Of course, not all jobs would be suitable for transformation into telecommuting, but probably there would be adaptable parts, and they will slowly increase thanks to redesigning of the jobs. For every job, especially those that are presential by nature, commitment to a team is needed, and that is at least partly the responsibility of every worker; that is the only way to create healthier job environments.

## 5. Conclusions

This paper provides evidence of the complicated relationships between job satisfaction and some of their antecedents. Presenteeism is positively related to job satisfaction. Meanwhile, overcommitment acts as a mediator in this relationship, and an overall positive effect of work-related bullying on job satisfaction is found. These relationships seem illogical, and they can only be explained by considering the flexibility of people’s perceptions: subjectivity may be more important than objectivity in explaining wellness. In these processes, the organization’s actions could be decisive in the improvement of workplaces.

## Figures and Tables

**Table 1:** Descriptive statistics.

	M	SD	VIF	Overcommitment	Presenteeism	Work-Related Bullying	Personal Bullying
Overcommitment	2.76	0.88	1.070	-			
Presenteeism	3.33	0.90	1.009	−0.063	-		
Work-related bullying	1.61	0.56	1.837	0.225 **	−0.084	-	
Personal bullying	1.26	0.41	1.830	0.226 **	−0.065	0.664 **	-
Job satisfaction	3.38	0.86		0.124 *	0.348 **	−0.218 **	−0.114 *

*Note:* * *p* < 0.05; ** *p* < 0.01.
